# Voltage-sensing domains: structural and functional diversity

**DOI:** 10.1007/s00249-025-01797-1

**Published:** 2025-09-13

**Authors:** Martin C. Heiss, Bernhard E. Flucher

**Affiliations:** 1 Department of Physiology and Medical Physics, Institute of Physiology, Medical University of Innsbruck, 6020 Innsbruck, Austria; 2 Department of Physiology and Medical Physics, Institute of Physiology, Medical University of Innsbruck, 6020 Innsbruck, Austria

**Keywords:** Voltage-sensing domains, Voltage-gated ion channels, gating charges, countercharges, charge transfer center, hydrophobic constriction site

## Abstract

Voltage-sensing domains (VSDs) are structural modules of voltage-gated ion channels, which sense changes in the membrane potential and, in response, open and close the channel’s ion conduction pore. VSDs comprise a bundle of four antiparallel transmembrane helices (S1–S4). Their basic function is well described by the sliding helix model. Upon membrane depolarization, the positively charged S4 helix slides upward and several of its positive gating charges cross the focused membrane electric field. This state transition is conformationally coupled to the opening of the channel gate. While this essential mechanism is common to all VSDs, different VSDs display a considerable structural and functional diversity, including the number of the gating charges, the nature of their countercharges, and the range, speed, and voltage dependence of the S4 movement upon activation. Here, we review these differences and discuss how they might function to determine the distinct gating properties of voltage-gated ion channels.

## Introduction

Voltage-sensing domains (VSDs) are structural modules of voltage-gated ion channels, which in response to membrane depolarization undergo conformational changes that open the channel gate. VSDs follow a stereotypic building plan of four transmembrane helices, expressed—four per channel—in all voltage-gated sodium (Na_V_), calcium (Ca_V_), and potassium (K_V_) channels as well as in the hyperpolarization-activated HCN channels (Jan 2025). Modified VSD variants are found in the non-voltage-gated TRP channels. A homologous VSD module is used to activate the voltage-sensitive phosphatase (Murata et al. 2005), and voltage-gated proton channels are formed of dimers of isolated VSD modules (Ramsey et al. 2006; Chanda and Bezanilla 2008). Thus, VSDs represent a very successful design element, primarily utilized to sense changes of the membrane potential in various membrane proteins. Over the past 30 years, many elegant studies using a broad range of techniques unraveled the basic building plan and general mode of function of VSDs. This literature has been covered by many excellent review articles, several of which will be cited below. Here, we direct our focus away from the commonalities of VSD function to their differences. We propose that the variations displayed in the structure of different VSDs underlie the diversity of their activation properties. Therefore, understanding how variations in structure cause functional diversity will reveal the molecular principles that tune the voltage sensitivity and kinetics of VSD activation to match the physiological requirements of their specific function. Given the author’s primary expertise, the choice of examples for the functional diversity of VSDs may be somewhat biased toward calcium channels. However, many notable examples from the sodium and potassium channel literature are given as well, demonstrating the general applicability of the functional principles.

Na_V_, low-voltage activated Ca_V_, and HCN channels function in cellular excitability (Hille 2001). They generate action potentials in excitable cells and propagate them along neuronal processes, through the myocard and into the depth of muscle fibers. K_V_ channels repolarize the cells and due to their highly diverse gating properties help generating a great variety of action potential shapes and firing patterns. Ca_V_ channels not only participate in the electrical signaling of excitable cells, but due to their ability to generate cellular calcium signals, they control many essential cell functions, including neurotransmission, synaptic plasticity, hormone secretion, and the contraction of heart and skeletal muscles. All these functions depend on the ion channel’s ability to sense changes in the membrane potential, endowed to them by their VSDs. However, the voltage range at which each channel activates differs greatly between channels, and so do the kinetics of voltage-dependent activation and inactivation of the channels. Consequently, the VSDs of all these channels essentially do the same thing, respond to changes of the membrane potential, but they do this at different voltages and with different speed. These functional differences must be encoded in the structural differences between the VSDs.

## The architecture of voltage-gated ion channels

Voltage-gated ion channels are tetramers or pseudo-tetramers with four VSDs controlling a single-channel pore. Each subunit or repeat consists of six transmembrane helices (S1–S6). The first four helices form the VSD. The S5 and S6 helices and the connecting extracellular loop form the pore domain (PD). Together four PDs form the ion conduction path with the selectivity filter on the extracellular side and the activation gate on the intracellular side. The activation gate is coupled to each of the four VSDs by the helical S4–S5 linkers, which transmits the action of the VSD to opening and closing of the gate (MacKinnon 2003; Catterall 2011; Huang et al. 2024).

**Fig. 1:**
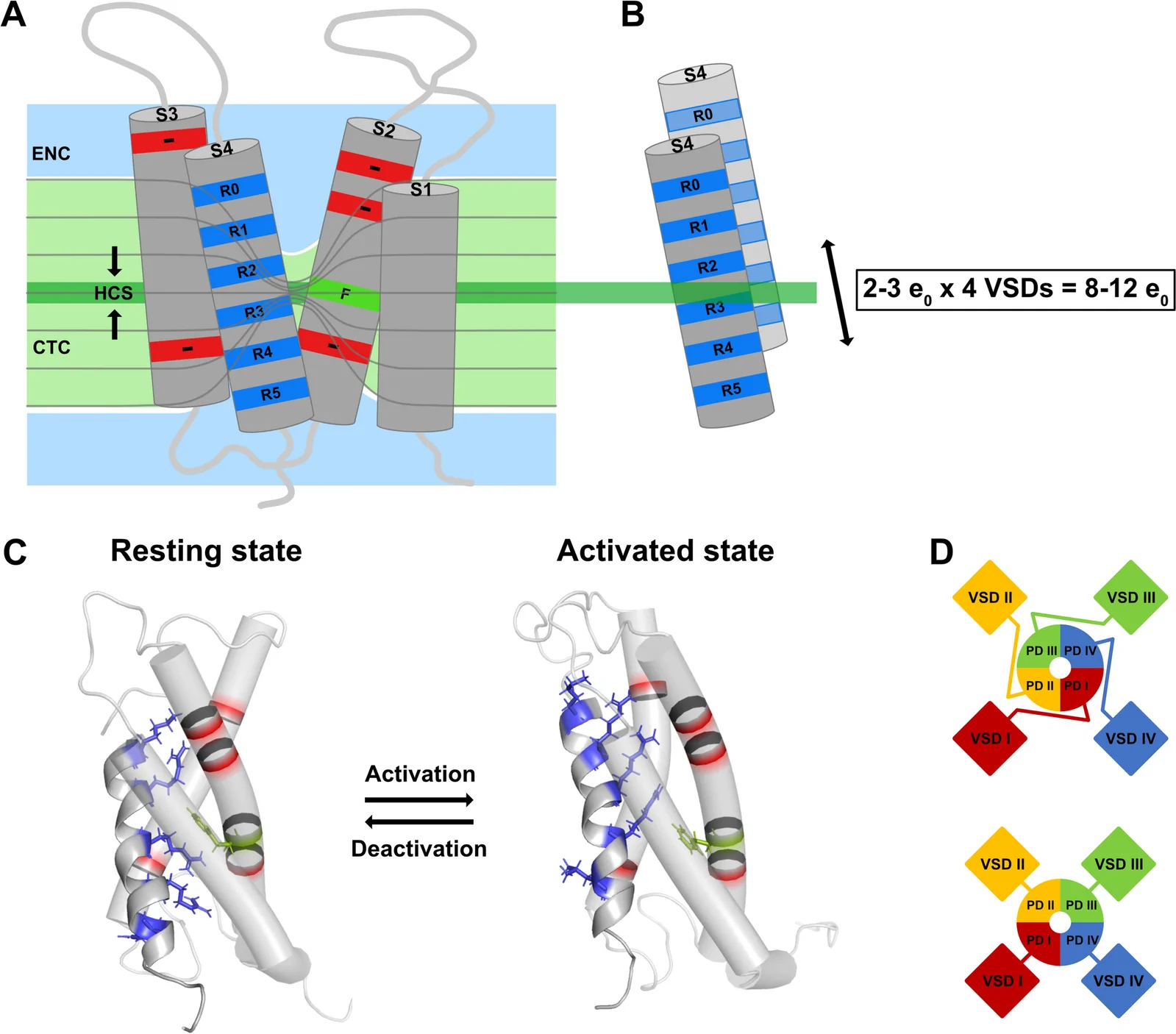
**VSD structures in activated and resting states**. **A** Schematic domain model of a VSD depicting the four transmembrane helices (S1, S2, S3, S4) with the positive gating charges (R0–R5) indicated in blue in S4 and countercharges of the extracellular negative cluster (ENC) and the charge transfer center (CTC) in red. The hydrophobic constriction site (HCS) including the conserved phenylalanine (F, green) focuses the electric field in the middle of the VSD and creates a narrow energy barrier for the transition of the gating charges between the intracellular and extracellular aqueous vestibules. **B** On membrane depolarization, typically 2–3 gating charges traverse the HCS, moving a total of 8–12 elementary charges per channel across the membrane electric field. **C** Ionic interactions between the gating charges (blue, arginine side chains) and countercharges stabilize the VSD in the resting and activated states and aid in the transition across the HCS. In the example shown (Ca_V_1.1 VSD I) at rest, only the outer two gating charges (K0, R1) are positioned above the HCS (indicated by the position of the phenylalanine; green). In the activated state, two additional gating charges (R2, R3) have moved to the cytoplasmic side of the HCS and only the innermost gating charge (R4) remains on the cytoplasmic side. **D** Voltage-gated ion channels contain four independent VSDs arranged around a common pore either in a domain-swapped (upper) or in a non-swapped configuration (lower). In the domain-swapped configuration, each VSD is positioned adjacent to the pore domain of the adjacent subunit (K_V_ channels) or repeat (Na_V_, Ca_V_ channels)

Different channel families present two fundamentally different modes of how the four VSDs are arranged around the pore and possibly how they functionally connect to it: the domain-swapped and the non-swapped arrangement (Fig. 1D) (Jan 2025). In domain-swapped channels, each VSD is arranged adjacent to the pore domain of the next subunit or repeat in a clock-wise manner; VSD I is positioned next to PD II, VSD II next to PD III, and so on. In channels with this arrangement, the electro-mechanical coupling is accomplished by the S4–S5 linkers, which span the distance between VSDs and PDs and together cradle the S5/S6 intracellular bundle crossing forming the activation gate. The domain-swapped arrangement is found in all Na_V_ and Ca_V_ channels as well as in the classical shaker-type K_V_ channels. EAG (“either-a-go-go or KCNH) potassium channels, HCN channels, and other relatives display a non-swapped arrangement with the VSD and PD of each subunit adjacent to each other. In these channels, other pathways for electro-mechanical coupling are being considered (Barros et al. 2020; Clark et al. 2020).

K_V_ channels and prokaryotic sodium channels form tetramers of four subunits, each containing one complete VSD and the two-helix PD. In homo-tetramers, the four VSDs contribute equally to channel gating. In heterotetrameric channels, the gating properties can be determined unequally by the properties of one or the other contributing subunit. Given the 40 K_V_ genes in humans, this allows for a huge number of possible combinations and constitutes a huge source of functional diversity. Although, in reality, the number of observed subunit compositions in K_V_ channels is much more restricted (González et al. 2012). Na_V_ and Ca_V_ channels, on the other hand, are products of a single gene with four homologous repeats, corresponding to the individual subunits of K_V_ channels (Catterall 2011). Consequently, they come only in a single combination of VSDs. Importantly, the four VSDs in these channels differ considerably from each other in structure and function, and they contribute to different degrees to channel gating, thus, constituting another source of functional diversity.

## The prototypic building plan of VSDs

VSDs are hourglass-shaped bundles of four antiparallel transmembrane helices (segments S1–S4) (Fig. 1A) (Long et al. 2005; Swartz 2008; Groome 2014; Catterall et al. 2017; Wang and MacKinnon 2017). The helices are connected by extracellular (S1–S2, S3–S4) and intracellular (S2–S3) loops of various sizes. The N- and C-terminal ends of VSDs are positioned on the cytoplasmic side of the membrane. The cytoplasmic end of S4 is hinged to the helical S4–S5 linker, which structurally and functionally connects the VSD to the channel’s pore domain (PD). The S4 helices typically contain four-to-six periodically spaced positively charged amino acids (arginines and lysines), each separated by two hydrophobic amino acid. The positive charges represent the gating charges onto which the membrane electric field exerts its electrostatic force, pulling the charged S4 inward in the polarized membrane, and releasing the pull and enabling the outward motion upon depolarization. At both sides, extracellularly and intracellularly, the helix bundle forms water-filled vestibules, into which the basic gating charges extend their side chains and form ion pair interactions with acidic countercharges in the adjacent helices. The two aqueous vestibules are separated by a ring of hydrophobic residues in the middle of the VSD, synonymously called the hydrophobic constriction site (HCS), hydrophobic plug, or gating pore. A highly conserved phenylalanine in S2 marks the HCS and together with two conserved negative countercharges, a glutamate in S2 and an aspartate in S3, represent a hallmark feature of VSDs. This motif has been called the charge transfer center (CTC), because it is thought to “catalyze” the transition of the gating charges across the HCS upon activation of the VSD (Tao et al. 2010).

## The sliding helix model

Over the last three decades, a host of biophysical and structural studies have led to a general agreement about the principal mechanism underlying VSD function. Whereas alternative models have been formulated, the sliding helix model (or helical screw model) (Catterall 1986; Durell and Guy 1992) is best supported by the sum of experimental data [for detailed reviews, see (Bezanilla 2000; Vargas et al. 2012; Catterall et al. 2017)]. According to this model, the positively charged S4 helix is moved up and down relative to the overall static S1, S2, and S3 helices by the changing polarity of the membrane potential (Fig. 1B). At rest, the attractive force of the negative membrane potential pulls S4 inward, so that all but the uppermost gating charges are accessible from the cytoplasmic side. Upon depolarization, S4 moves outward and two-to-three gating charges that at resting potentials were located on the cytoplasmic side, now are accessible from the extracellular side. In all but the hyperpolarization-activated HCN channel, the down-position corresponds to the resting state and the up-position to the activated state. Entry into the activated state favors the opening of the channel gate.

Upon this state transition, several gating charges cross the membrane electric field, which is focused at the HCS. This up and down movement of charged arginine and lysine side chains across the electric field can be recorded as capacitive currents, called the on- and off gating currents, respectively (Armstrong and Bezanilla 1973; Schneider and Chandler 1973). The recorded charge moved across the electric field matches the experimentally determined transition of two-to-three gating charges per VSD. Moving charged amino acids across the hydrophobic plug in the transition pathway of the S4 helix is energetically unfavorable. However, charge transition is facilitated by the limited thickness of the HCS, estimated to be near 4 Å (Ahern and Horn 2005). In addition, the transition of the gating charges across the HCS is supported by their transient interactions with the residues of the CTC and possibly further countercharges on both sides of the HCS (Tao et al. 2010) (Fig. 1C). The sum of interactions between the gating charges and countercharges also stabilizes the VSD in its consecutive resting and activated states (Groome and Winston 2013). Most current models envision one or more intermediate states between the lowest resting and fully activated states, according to the number of gating charges transitioning across the HCS. Indeed, gating currents can be recorded during the transition between closed states (Bezanilla 2018). Some experiments also suggest multiple activated, inactivated, and relaxed VSD states (Catterall et al. 2017). However, the definition of state very much depends on the experimental paradigms and models used in the respective studies.

The recent structural revolution, particularly with the advances in cryo-electron microscopy (cryo-EM) and computational structure modeling, greatly boosted the structure–function studies of ion channels and of VSDs in particular (Huang et al. 2024). While, ironically, the earliest achieved X-ray structures of a voltage-gated channel produced misleading information on VSD structure (Jiang et al. 2003), the bulk of currently available structures is in good agreement with the predictions of the sliding helix model. To date, the greatest limitation still results from the fact that most biochemical preparations used for structure determination rely on channels from depolarized membranes and thus yield channel structures in the activated, or more likely an inactivated or relaxed state, with the VSD in the up-position and the gate closed. To overcome this problem, researchers are applying a multitude of preparations and all kinds of tricks to obtain channels in the resting state. Recently, several of these efforts were successful, and the number of VSD structures in putative resting and intermediate states is rapidly increasing (Xu et al. 2019; Wisedchaisri et al. 2019, 2021; Gao and Yan 2020; Gao et al. 2023a). At the same time, advances in computational structure modeling help to fill the gaps in the state transitions and they allow us to actually study the dynamics of VSD movement and its functional coupling to the gate (Jensen et al. 2012; Yarov-Yarovoy et al. 2012; Delemotte et al. 2015; Elhanafy et al. 2025). Together, these approaches provide valuable support to the current models of channel function. At the same time, they reveal differences between channels and unexpected aspects that raise new challenges for structure–function analyses and further advance existing models of VSDs.

## Obligatory vs. allosteric gating models

Finally, having four VSDs controlling a single-channel pore allows for different modes of cooperation to accomplish this task. According to the pure obligatory model, all four VSDs need to be in the up-state (activated) for the channel to open (Hodgkin and Huxley 1952; Zagotta et al. 1994; Bezanilla et al. 1994). For example, in a Shaker K_V_ channel concatemer with three of the four VSDs mutated to position in the up-state, the activation of the fourth VSD was necessary for gating of the channel (Gagnon and Bezanilla 2009). A variation of the obligatory model would allow for a smaller number of VSDs to be necessary for channel opening, but still in an all or none fashion; i.e., the channel only opens, when all of the required VSDs are in the activated state. For example, in Na_V_1.4, the three rapidly moving VSDs I, II, and III need to be in the up-state to allow channel opening, whereas the slowly moving VSD IV reaching the up-state allows the channel to enter the inactivated state (Capes et al. 2013; Ahern et al. 2016). In the obligatory model, voltage dependences of activation and deactivation are strictly limited by the VSD with the most right-shifted voltage dependence. On the other hand, activation kinetics would be determined by the slowest VSD, whereas the kinetics of deactivation is determined by the fastest VSD, because the channel opens only after the last VSD has reached the activated state and closes as soon as the first VSD exits the activated state.

Alternatively, channel opening can be controlled by the VSDs allosterically (Horrigan and Aldrich 2002; Sigg 2014). According to the allosteric model, activations of all four VSDs contribute toward an increase in open probability. With all four VSDs in the up-position, the open probability is highest. However, the channel can also open with some VSDs in the down-position and, at least theoretically, even without any of the VSDs in the activated state. For example, a recent study demonstrated that a concatemer of KCNQ2 (K_V_7.2) with three of the four subunits locked in the resting state was able to activate currents with similar voltage dependence and kinetics as wild-type controls, albite with reduced single-channel conductance (Hinojo-Perez et al. 2025). The contributions of the individual VSD toward channel gating (i.e., the energy contributed toward channel opening) can differ between the four VSDs (Marks and Jones 1992; Pantazis et al. 2014; Beyl et al. 2014). Accordingly, also the individual VSD’s contributions of specific activation properties, like its voltage dependence and kinetics of activation, would vary.

In addition to the state transitions of the four VSDs (resting to activated), many models include another concerted state transition for the actual gating of the pore (closed to open) (Sigworth 1994; Hille 2001; Chowdhury and Chanda 2012). This electro-mechanical coupling process typically accounts for an additional right-shift of voltage dependence or a delay in activation not directly related to the properties of the VSDs alone. Furthermore, it is consistent with the observed effects of mutations in the gate, which alter the voltage dependence and kinetics of channel gating independently of the properties of the VSDs.

Which of the models best explains experimental data differs from channel to channel and often from one experimental paradigm to another. Therefore, the two models appear not to be mutually exclusive. Rather, channel activation by the four VSDs differs from channel to channel and may well combine obligatory and allosteric elements. For example, voltage gating of the calcium channel Ca_V_1.2 is best described with an allosteric model in which the four VSD differentially contribute to pore opening (Pantazis et al. 2014). Nevertheless, allosterism appears to be limited to the interaction with the common gate and does not extend to the interactions between the VSDs, as modulation of the properties of individual VSDs does not affect the properties of the other VSDs (Savalli et al. 2016; Nilsson et al. 2024) (but see Chanda et al. 2004).

## The diversity of VSD structure and function

**Fig. 2:**
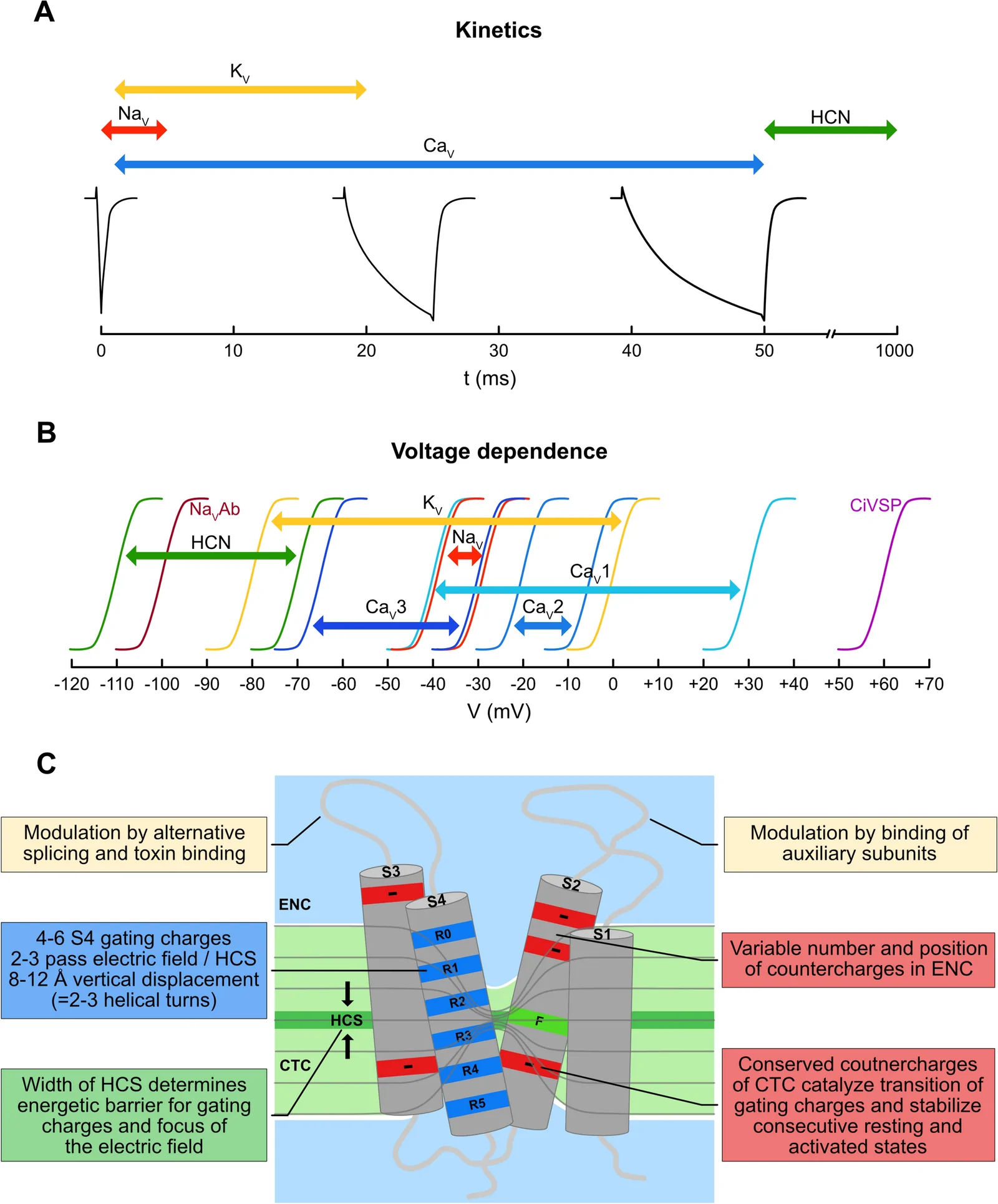
**Diversity of gating properties of voltage-gated ion channels and of VSD structures contributing to the functional variability**. Ranges of activation kinetics (**A**) and voltage dependence of activation (**B**) of the different families of voltage-gated ion channels and CiVSP, with schematic drawings of exemplary inward cation currents (**A**) and *Q*/*V* curves (**B**). **C** Trans-membrane domain structure of a VSD with S4 gating charges indicated in blue, countercharges of the ENC and CTC indicated in red, and the conserved phenylalanine in the HCS indicated in green. Variability in these structures and their interactions during the state transitions give rise to the distinct activation properties of VSD and thus contribute to the diversity of channel gating properties. Moreover, modulation of these interactions by toxins or subunits binding to the extracellular loops further adds variability to VSD function

Functional analyses of voltage-gated ion channels revealed a considerable variety of gating properties. Sodium channels, responsible for triggering action potentials, typically activate rapidly and at lower membrane potentials than potassium channels, which are responsible for repolarization. Calcium channels, involved in a broad range of cell signaling functions, activate over a wide range of voltage potentials and show greatly diverse activation and inactivation kinetics (Fig. 2). Channelopathies caused by mutations, which alter these parameters even slightly, impressively demonstrate the importance of the exact tuning of gating properties (Mantegazza et al. 2021). What are the molecular mechanisms by which the VSDs of these channels accomplish this great diversity of gating properties?

Whereas the pore domain with the activation and inactivation gates, as well as modulation by auxiliary subunits also contribute to the gating parameters, the VSDs as primary voltage sensors are the principle agents of voltage-dependent ion-channel gating. Particularly, the voltage dependence and kinetics of channel activation can be ascribed directly to the properties of the voltage- and time-dependent state transitions of the VSDs. But also the properties of voltage-dependent inactivation and the channel open probability can fully or in part be controlled by the actions of the VSDs. Thus, the great diversity of these biophysical channel properties is indicative of a similarly large diversity of VSD structures and functions. The large number of voltage-gated ion-channel genes—in humans 40 K_V_ channels (Gutman et al. 2005), 10 Na_V_ channels (Catterall et al. 2005b), and 10 Ca_V_ channels (Catterall et al. 2005a), plus multiple splice variants thereof—allows for a large genetic and structural diversity of VSDs. Nevertheless, the overall protein fold and key elements like the positive charges in S4, the HCS, and the countercharges of the CTC follow a highly stereotypic building plan and suggest a uniform functional principle. Although this is true in general terms, VSDs differ between different ion channels, as well as among the four VSDs of individual Na_V_ and Ca_V_ channels. While the extent of non-conserved residues in VSDs easily explains their structural and functional diversity, finding the molecular principles by which the functional properties are tuned to the specific need of each channel type poses a substantial challenge for current ion-channel research.

## S4 gating charges

The most striking feature of the VSD is the gating charges regularly spaced at a distance of three residues in the S4 helix. They represent the voltage sensor proper and move up and down through the membrane electric field in response to changes in the membrane potential. They also represent the electrical charge measured as the on- and off gating currents. Without these gating charges, a VSD cannot sense voltage changes. However, the number of gating charges differs substantially between channels and even among the four VSDs of a single heterotetrameric channel (Groome and Bayless-Edwards 2020). For instance, the S4 helices of the bacterial Na_V_Ab channel as well as the first VSD of the mammalian Na_V_1.4 each contain four gating charges, while the fourth VSD of Na_V_1.4 contains eight regularly spaced arginines and lysines in its S4 helix. Most K_V_ channels contain six gating charges; with K_V_1.1 and 1.2 possessing a seventh, and K_V_2.1 and 4.2 missing the uppermost positive charge in their S4 helices. The number of gating charges in Ca_V_ channels ranges between four and six in the four VSDs, with differences both between the four VSDs of one channel and, to a lesser degree, between the same VSDs of different channel isoforms.

### Theoretical considerations

Theoretically, the larger the number of gating charges is, the higher the voltage sensitivity will be. Indeed, many studies in which gating charges have been replaced with uncharged amino acids report a decreased slope of the voltage dependence of activation, indicative of a reduced voltage sensitivity. However, this is not consistently observed, suggesting that not all gating charges in all VSDs contribute equally to the voltage dependence of current activation (Islas and Sigworth 1999). In reality, the action of gating charges depends on the shape of the electric field and on the actual displacement of the S4 helix upon depolarization and repolarization; i.e., on the number of gating charges traversing the electric field. The electric field is not uniform across the thickness of the membrane, but concentrated at the HCS in the center of the VSD (Ahern and Horn 2005; Delemotte et al. 2015; Islas 2016). Therefore, charged residues close to this focus will experience a stronger electrostatic force than charges distant from this focused electric field. Because the thickness of the HCS, where the electric field is densest, is estimated at about 4–8 Å (i.e., merely about a tenth of the thickness of the lipid bilayer), only two gating charges separated by one helical turn of 5.4 Å will reside in this focus area in any state of the VSD. Of course, as the S4 helix moves up and down upon activation and deactivation, the specific gating charges within the focused electric field will sequentially exchange. Considering a vertical displacement of about 8–12 Å only about four different gating charges will eventually reside in the vicinity of the focused electric field and only 2–3 of them will pass through it entirely.

### Functional evidence

These approximations are consistent with data from mutagenesis experiments directly probing the transfer of S4 gating charges across the HCS (Yang et al. 1996; Starace and Bezanilla 2001; Ahern and Horn 2004), as well as with estimates of the elementary charges per channel moved through the electric field. The steep voltage dependence of current activation suggested the movement of about 12 elementary charges per channel (Sigworth 1994). Early analyses of gating currents determined the effective charge necessary to open a channel. In shaker K_v_ channels, it was 13 elementary charges e_0_ per channel, in Na_V_1.4 12 e_0_ and in the Ca_V_2.3 channel 9 e_0_ (Hirschberg et al. 1995; Aggarwal and Mackinnon 1996; Seoh et al. 1996; Noceti et al. 1996; Börjesson and Elinder 2008). These numbers of charges moved during channel gating correspond to approximately three (K_V_, Na_V_) and two (Ca_V_) gating charges per VSD crossing the membrane electric field. Thus, despite the considerable variability in the number of gating charges found in S4 segments of different VSDs, the number of charges moved across the membrane electric field is fairly uniform. Consequently, a larger number of S4 gating charges will not necessarily result in a higher voltage sensitivity of a VSD (Islas and Sigworth 1999). Although, data from the highly diverse potassium channel families indicate that to some extent this may be the case (Jan 2025). Even if a VSD with many gating charges can travel a larger distance and involve more of its gating charges in the state transitions (at least theoretically), in any given state, the same limited number of gating charges will experience the full force of the electric field

### Structural evidence

Structure analyses of various voltage-gated ion channels corroborates this notion. Due to the depolarized state of the biochemical preparations used to isolate voltage-gated ion channels, their structure typically is solved in a state with the VSDs in the up-position and the pore closed. Probably, these structures resemble an inactivated state of the channel, but the VSDs themselves are in their activated up-state. Therefore, it should be fairly straight forward to answer the question as to which gating charges reside above, and which below the HCS in the activated state, and how uniform this configuration is among the different channels. In practice, such a comparison is complicated by the lack of agreement on which state these structures represent as well as by the different numbering of gating charges used in the various channels and studies. For better comparability, here we use the prokaryotic sodium-channel Na_V_Ab with its minimal number of four gating charges as reference, to which we align the sequences and numbering of other VSD.

Na_V_Ab has been the first member of the Na_V_/Ca_V_ family to be crystalized and its structure solved using X-ray crystallography (Payandeh et al. 2011). Presumably, it represents an ancestral channel to both the members of the eukaryotic Na_V_ and Ca_V_ channel families (Catterall et al. 2017). It is a homotetrameric channel with a domain-swapped arrangement and its S4 helix contains only four gating charges (all arginines). These four gating charges, numbered R1, R2, R3, and R4, represent the minimal functional set of gating charges in native VSDs of voltage-gated ion channels. They correspond to the gating charges R2 to K5 in S4 helices of K_V_ channels, which have been shown to move across the membrane electric field (Bezanilla 2000). Recently, the structure of Na_V_Ab has been solved in a putative resting state (Wisedchaisri et al. 2019), thus providing us with the structural endpoints of the VSD in the polarized and depolarized membrane electric field. In the activated state of Na_V_Ab, R1, R2, and R3 reside above, and R4 below the HCS, latched in the CTC. In the resting state of Na_V_Ab, only R1 is positioned above the conserved phenylalanine of the HCS. A matching configuration of gating charges has been found in individual VSDs of Ca_V_2 channels trapped in the resting state (Gao et al. 2021, 2023b; Yao et al. 2022b). Accordingly, R1 through R4 seem to represent the minimal structural unit involved in the voltage-sensing action, but only R2 and R3 fully traverse the HCS.

This notion is supported by structures from several Ca_V_ and Na_V_ channels (Wu et al. 2015; Pan et al. 2018; Yao et al. 2022b; Gao et al. 2023b). In the activated state, the phenylalanine marking the HCS is consistently positioned between R3 and R4, even in VSDs containing an extra R5 on the cytoplasmic side. The available resting sate structures show R1 close to but still above the HCS, while R2–R5 reside on the cytoplasmic side of the HCS. Whether in Ca_V_ and Na_V_ channels, an additional gating charge on the extracellular side (K/R0) would allow S4 to move further down currently remains undecided, because to date resting-state structures of VSDs containing K/R0 have not been solved. In the shaker K_V_ channel, this actually might be the case (Starace and Bezanilla 2001; Broomand et al. 2003; Campos et al. 2007; Henrion et al. 2012; Delemotte et al. 2015). Moreover, extensive experimental and structural data indicate that the large structural and functional diversity of potassium channels is accompanied by a considerable variability of gating charge movements in response to depolarization. The pertinent experimental evidence for the voltage sensor actions in the different potassium channel families has been compiled in great detail in a recent review article by Jan (2025). For the K_V_ channels, she summarizes that voltage sensor activation via depolarization might involve the translocation across the CTC of four arginines in K_V_1 and three in K_V_2, K_V_3, and K_V_4 channels. Though, the translocation of four gating charges per K_V_1 subunit would exceed the experimentally determined 13 e_0_ per shaker channel tetramer (Aggarwal and Mackinnon 1996; Noceti et al. 1996).

### Supplementary actions of gating charges

At this time, the importance of the different number of gating charges is still not fully understood. The notion that a longer S4 helix would result in a larger range of movement cannot be generally applied (Yang et al. 1996; Islas and Sigworth 1999). Alternatively, additional gating charges, which do not pass the HCS, may contribute to the stabilization of particular states by interacting with countercharges in the nearby helices of the VSD or with the negatively charged head groups of the membrane lipids. Thus, the effect of the gating charges on gating properties heavily depends on the environment in which they operate, including parameters like the thickness of the HCS and the countercharges and other interaction partners in the VSD (see below). Moreover, the two amino acids forming gating charges, arginines and lysines, are not functionally equivalent. Depending on the nature of their interaction partners and the molecular interactions formed, one or the other may result in stronger interactions (Tao et al. 2010). Therefore, whether arginine or lysine forms a given gating charge adds another level of molecular diversity determining the activation properties of a VSD.

### Evidence from mutagenesis studies

A gating mechanism by which the negative membrane potential at rest pulls positive gating charges inward and the reversed direction of the electrostatic force upon depolarization pulling S4 outward is highly intuitive. Moreover, ionic interactions of positive gating charges with negative countercharges facilitating the charge transfer across the HCS and involved in stabilizing consecutive states of the VSD are similarly intuitive. However, experiments show that this picture is a gross oversimplification. As expected, replacing all gating charges of a VSD with uncharged amino acids disables voltage sensing. However, how this affects channel function depends on several factors. Presumably, a completely charge-neutralized VSD is in the up-position (i.e., no longer pulled into the resting state by the membrane potential). In non-inactivating channels, operating in an obligatory mode, one or more VSDs trapped in the up-state may facilitate channel activation by the remaining functional VSDs (Gagnon and Bezanilla 2009). In inactivating channels, trapping a VSD in the up-state might send the channel into slow inactivation (Beyl et al. 2012). Moreover, VSDs of some channels seem to be non-essential for current activation. Consequently, incapacitating them by neutralizing their gating charges remains without functional effect to channel gating (Beyl et al. 2016).

### The nature of the gating charge interactions

Whereas neutralizing all gating charges of a VSD usually results in the loss of its function, the outcome of partial neutralization experiments is quite variable and not always as expected. Replacement of S4 arginines by citrulin, an isosteric but neutral analog of arginine, resulted in much smaller shifts in the voltage dependence of activation than substitutions of the same gating charges with uncharged natural amino acids. This demonstrates the importance of size and shape in addition to that of the positive charge (Infield et al. 2018). Most unexpectedly, in a remarkable study by Díaz-Franulic et al. (2018), the reversal of the gating charges (substitutions of positively charged arginines by negatively charged aspartates) did not result in the loss of voltage-sensing function, but allowed channel function with almost normal gating properties. A possible interpretation of this conundrum is that the functionally important interactions between gating charges and countercharges are not ionic bonds requiring a pair of oppositely charged side chains, but water-mediated ionic interactions, which can be established between pairs of equally charged moieties. Thinking of it, such water-mediated interactions may better serve the transient and dynamic nature of such bonds, which rapidly form and brake upon the consecutive state transitions of a VSD. Together, the experimental evidence confirms the central importance of the gating charges for the function of VSDs. Variations in their number, type, and their interactions with the environment all contribute to the diversity of VSD properties and function.

## Thickness of the hydrophobic constriction

As described above, the seal between the aqueous extracellular and intracellular compartments and thus the focus of the membrane electric field is the HCS in the center of the VSD. This barrier not only prohibits the flow of soluble ions between the two compartments, but equally establishes an energetic barrier for the transition of the gating charges across the hydrophobic plug. Consequently, its thickness affects both the resistance to charge transition (the thicker, the higher the resistance), and the strength of the local electric field (the thinner, the more focused, the stronger). Therefore, a very thin HCS best serves the purpose of the voltage sensor. It minimizes the size of the energetic barrier and maximizes the electrostatic force acting on the gating charges.

The HCS is made up of multiple hydrophobic residues in the three helices (S1, S2, S3) flanking S4. The phenylalanine in S2 is highly conserved and represents one of the hallmark features of VSDs. Additional hydrophobic residues in the middle of the S1 and S3 are also found in all voltage-gated channels and together with the S2 phenylalanine form the gasket separating the inner and outer aqueous vestibules (Campos et al. 2007; Lacroix and Bezanilla 2011). However, these differ between VSDs. Variability of these hydrophobic residues has been suggested to determine the kinetic differences between ion-channel families and between individual VSDs of heterotetrameric channels. Additional hydrophobic side chains in these positions may increase the thickness of the HCS. Consequently, differences between VSDs in the number and hydrophobicity of these residues will influence the barrier through which the gating charges have to move, the electrostatic force of the focused electric field exerted on the gating charges and ultimately the energy barriers between the consecutive states. Molecular dynamics simulation confirms that the stronger dielectric polarization by threonine as opposed to isoleucine can explain the kinetic differences between Na_V_ and K_V_ channels, respectively (Catacuzzeno et al. 2021). Thus, variability in the width of the HCS is a determinant of the kinetic differences between VSDs and ion channels, and accordingly has been ascribed as “speed-control site” (Lacroix and Bezanilla 2011; Lacroix et al. 2013).

## Countercharges

To cross the energetic barrier of the HCS the positively charged gating charges need some help, and this help may come in the form of negative countercharges with which the gating charges can form ion pairs as they transit from the resting down- to the activated up-position (Catterall et al. 2017). Countercharges involved in interactions with the S4 gating charges have been described both, on the extracellular and the intracellular side of the HCS, called extracellular negatively charged cluster (ENC) and intracellular negatively charged cluster (INC), respectively. Early charge-reversal and charge-swapping studies in potassium channels, and cysteine substitution and disulfide-locking studies in bacterial sodium channels established sequential pair-wise interactions of such countercharge with the gating charges (Decaen et al. 2009). These experiments demonstrated the importance of transient formation of these ion pairs in the assembly of functional channels and in mediating the conformational transitions from the resting to the activated state (for a comprehensive review see Groome and Bayless-Edwards 2020).

### The charge transfer center (CTC)

Two highly conserved negative residues—a glutamate in S2 one helical turn below the phenylalanine marking the HCS and an aspartate in the middle of S3—have been implicated in a catalytic function for the S4 transition into the activated state. Together with the conserved phenylalanine, they have been termed charge transfer center (Tao et al. 2010). Additional, charged residues of S1, S2, and S3 mainly in the ENC but also in the INC have been shown to function as countercharges with important functions in the state transitions underlying activation and inactivation of the channels. As opposed to the two negative charges of the CTC, which belong to the INC, all other putative countercharges are not highly conserved between channels nor between the four VSDs of pseudo-heterotetrameric ion channels (Palovcak et al. 2014). Therefore, the different types, numbers, and positions of countercharges established in different VSDs represent a rich source of structural and functional variability. Or, put in other words, the conserved CTC is the common theme of VSDs, whereas the additional countercharges are the variation to this theme. The two clusters of countercharges also differ in their mode of function. As expected for countercharges, the functional effects of mutating charged residues in the ENC are contingent on charge. Surprisingly, however, using substitutions with unnatural amino acids in the shaker K_V_ and in Na_V_1.4, Pless and colleagues showed that the functions of the CTC countercharges do not depend on their negative charge (Pless et al. 2011, 2014). Apparently, their interactions with the gating charges are not mediated by electrostatic attraction of the oppositely charged side chains, but may be mediated by water molecules in the inner aqueous vestibule.

One way or another, the countercharges in the INC and the ENC position the gating charges in close proximity of the HCS and thus facilitate their transition across this high but narrow energetic barrier as S4 is pulled up and down by the electrical potential. In the course of this transition, a transient interaction of the passing gating charge with the aromatic ring of the conserved phenylalanine probably plays an essential role. Exchanging the phenylalanine with other aromatic amino acids and combining them with either arginine and lysine gating charges revealed that the relative strength of their interactions modulates the activating or deactivating transitions of the VSDs (Tao et al. 2010). In addition, these experiments in the shaker K_V_ channel indicated that entry of the innermost gating charge into the occluded binding site composed of the CTC, prompts pore opening and that the strength of this interaction contributes to stabilizing the activated state. It is reasonable to assume that this stabilizing function not only applies to the innermost gating charge in the S4 up-state, but equally to other gating charges transiently engaging in the CTC in the resting and intermediate states. Thus, the CTC appears to serve two functions: Catalyzing the transition of gating charges across the HCS and stabilizing them in the consecutive states. Functionally, these actions are reflected by changes in the speed and voltage dependence of activation, respectively. Because the three components of the CTC are conserved among VSDs, this function probably is ubiquitous in all VSDs.

### Countercharges of the ENC

**Fig. 3:**
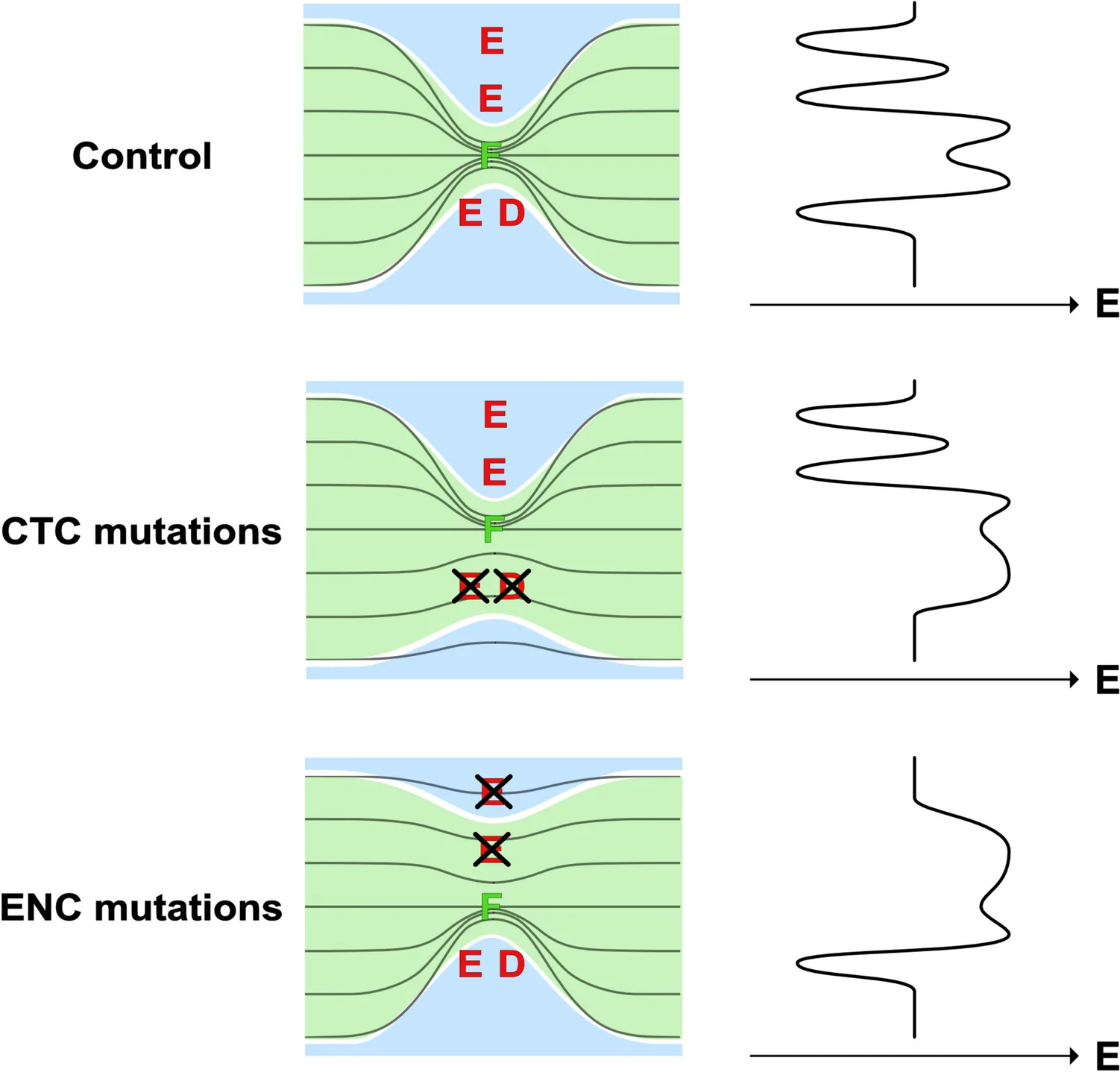
**Role of countercharges in shaping the energetic barrier for gating charge transfer across the HCS**. Left: model depicting interaction partners of the gating charges lining the gating pathway of the S4 helix through the VSD. Hydrophilic (blue) and hydrophobic regions (green) with membrane electric field lines focused at the HCS. Right: hypothetical 2D energy plot for interaction energies of gating charges with their interaction partners, and changes thereof upon mutation of inner (CTC) or outer (ENC) countercharges. The balance between hydrophobic residues in the middle and the charged and polar residues on either side of the HCS determines the focus of the electric field and the energy barrier for gating charge transfer. Transient cation-π interactions with the aromatic ring of the phenylalanine curb the energy barrier and catalyze the transition of gating charges across the HCS without much stabilizing this transition state itself. The negative countercharges create energy wells, which stabilize gating charges as they sequentially pass from the resting to the activated state. Mutations of countercharges on either side are expected to change the width of the HCS, the focus of the electric field, and they eliminate energy wells stabilizing gating charges in resting or activated states

Many other charged and polar amino acids in S1, S2, and S3 have been suggested to function as countercharges, which interact with the S4 gating charges in one or more states. These vary greatly between VSDs of the same and different channels, both in structure and function (Groome and Bayless-Edwards 2020). Substitution of these countercharges with uncharged amino acids frequently affects the voltage dependence of activation. Replacement of countercharges involved in the stabilization of the activated state leads to a right shift toward more positive potentials. On the other hand, a hyperpolarized, left-shifted, voltage dependence of activation upon charge removal indicates a role of the countercharge in stabilizing a resting state. Energetically, altered voltage dependencies can be envisioned as changing the depths of energy wells; the deeper, the more stable the state will be (Fig. 3). On the contrary, changes of activation kinetics can be viewed as changes of energy barriers. Heightened energy barriers slow down the transition between states and consequently increase the total time it takes for S4 for reaching the up-state.

**Fig. 4:**
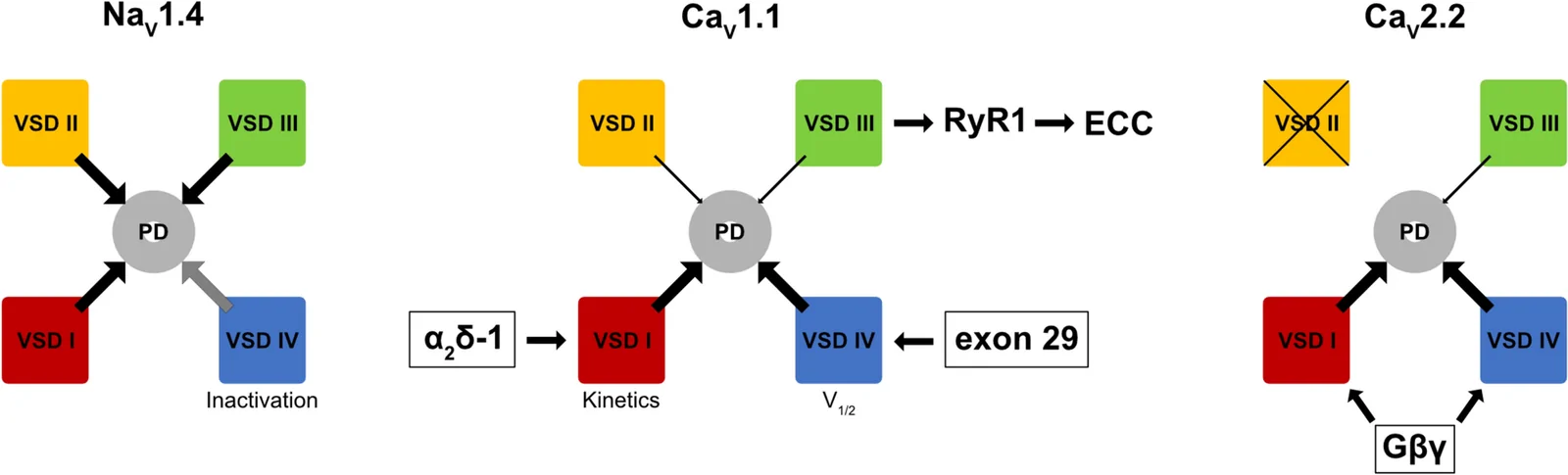
**Examples of differential functions and contributions of the four VSDs in controlling channel gating**. Gating of the Na_V_1.4 channel requires the rapid activation of three of its VSDs (I, II, III), whereas delayed activation of the fourth (VSD IV) is necessary for fast channel inactivation. Ca_V_1.1 channel gating is primarily controlled by two VSDs (I and IV), which determine specific gating properties. VSD I controls the slow activation kinetics, which is further modulated by its interaction with the auxiliary α_2_δ-1 subunit. VSD IV regulates the voltage dependence of activation by alternative splicing of exon 29. VSD III of Ca_V_1.1 is the sole regulator of skeletal muscle EC coupling (by its conformational coupling to RyR1) and its activation is further necessary for channel gating. VSD II contributes little to channel gating in Ca_V_1 channels and even remains locked in the resting state in activated Ca_V_2 channels. Specific interactions of G-protein βγ subunits with VSDs I and IV switch the Ca_V_2.2 channel into a reluctant gating mode 2 leading to inhibition

The large number and considerable variability between VSDs precludes addressing all identified countercharges. A comprehensive overview of the state of the art has been given by Groome and Bayless-Edwards (2020). In Fig. 4 of that review, they show the countercharges identified in a network analysis from short time-scale molecular dynamics simulations of the four VSDs of Na_V_1.4 in the resting, an intermediate and the activated state. This exemplifies several important points about the nature of gating charge–countercharge interactions, which probably apply to pseudo-heterotetrameric channels in general: The glutamate (S2) and aspartate (S3) of the CTC exist in all four VSDs and interact with gating charges in all states. Other countercharges on both sides of the CTC differ between the four VSDs and engage in state-dependent interactions with the gating charges. Consequently, the number of interactions established in each state differs between VSDs. Some form more interactions in the activated state, others in the intermediate or resting states. Considering these diverse patterns of ion pairs, it is easy to envision how they may be differentially stabilizing specific states and consequently differentially regulating the activation properties of each VSD. However, when looking for possible explanations for the well-known functional specialization of the Na_V_1.4 VSDs—the slow activation of VSD IV—this network analysis does not provide any obvious clues.

In terms of functional heterogeneity, the Ca_V_ channel family is the most striking example. Ca_V_ channels activate at voltages between − 60 mV (Ca_V_3.1) to + 40 mV (Ca_V_1.1) and with kinetics in the range of milliseconds and hundreds of milliseconds, respectively (Fig. 2A, B). Analysis of individual VSDs using voltage-clamp fluorometry and molecular structure modeling indicate that these distinct gating properties actually are the consequences of greatly different activation properties of the VSDs (Pantazis et al. 2014; Fernández-Quintero et al. 2021; Pelizzari et al. 2024). A series of mutagenesis studies in the skeletal muscle Ca_V_1.1 revealed several mechanisms by which the interactions between gating charges and countercharges determine the specific properties of VSDs and thus the gating properties of the channel. In this channel, the countercharges of the ENC differ substantially between the VSDs. In VSD I, three functionally relevant countercharges in S2 and S3 have been characterized. In VSD IV, a single countercharge in S3 plus one outside the VSD in the adjacent pore domain of repeat I interact with the outer gating charges (Tuluc et al. 2016; Fernández-Quintero et al. 2021; El Ghaleb et al. 2021). Mutagenesis experiments demonstrated that all these interactions in the ENC of VSDs I and IV regulate the voltage dependence of activation, presumably by stabilizing the VSD’s activated state. This function is complemented by the concurrent interaction of the innermost gating charge with the CTC, which further stabilizes the activated state (Pelizzari et al. 2024; Heiss et al. 2025). Surprisingly, negatively charged amino acids in the corresponding positions of VSDs II and III have little impact on the voltage dependence of activation (Costé de Bagneaux et al. 2018); most likely, because these VSDs do not significantly contribute to channel gating (Savalli et al. 2021). In VSD I, ENC interactions are further important for regulating the activation kinetics of the channel. The sequential formation and breaking of interactions of multiple gating charges with the ENC slow down the upward S4 movement and thus determine the extremely slow activation characteristic for Ca_V_1.1 (Fernández-Quintero et al. 2021; Heiss et al. 2025). This example highlights how the diversity of interactions between the gating charges and the countercharges leads to distinct activation properties of VSDs and thereby shapes the specific gating properties of different channels.

## Gating charge–ENC interactions involved in channel modulation

Intriguingly, gating charge–countercharge interactions also serve as a mechanism for the modulation of calcium channel properties. For example, insertion of an alternatively spliced exon resulting in a 19 amino-acid expansion of the S3–S4 linker in VSD IV of Ca_V_1.1 disrupts the interactions between the outer gating charges and the ENC and thus destabilizes the activated state (Tuluc et al. 2009, 2016). Consequently, the adult isoform of Ca_V_1.1, containing exon 29, has a 30 mV right-shifted voltage dependence of activation and several-fold reduced current density. Alternative splicing is found in equivalent positions of other L-type calcium channels, where this mechanism might serve similar modulatory functions (Costé de Bagneaux et al. 2018). Moreover, the extracellular S3–S4 linkers of several other voltage-gated ion channels contain binding sites for channel modulating toxins. Importantly, such toxins acting at the S3–S4 linkers specifically ablate the actions of only the VSD to which they are binding. For example, sodium channel toxins binding to S3–S4 of VSD II stabilize it in the up-state and specifically affect the voltage dependence of activation. In contrast, toxins binding to the S3–S4 loop of VSD IV prevent fast inactivation, without altering activation properties. Because fast inactivation requires prior activation of VSD IV, these toxins probably lock this VSD in a resting state (Stevens et al. 2011). In analogy to the mechanism causing the effects of S3–S4 alternative splicing, also toxin binding to this modulatory site in the S3–S4 loop might affect the interactions between the gating charges and the ENC countercharges and thereby cause the stabilization or destabilization, respectively, of the VSD’s activated state.

Furthermore, modulation of channel properties by auxiliary subunits might act on gating charge–countercharge interactions. For example, the auxiliary α_2_δ-1 subunit is a modulator of high-voltage-activated calcium channels. In the cardiac/neuronal Ca_V_1.2, it left-shifts the voltage dependence of activation and accelerates activation kinetics (Tuluc et al. 2007). In contrast, in the skeletal muscle Ca_V_1.1, the α_2_δ-1 subunit contributes to the channel’s typical slow activation (Obermair et al. 2005). This suggests that α_2_δ-1 functions by stabilizing the intrinsically fast or slow properties of these two channel isoforms (Obermair et al. 2008). Voltage-clamp fluorometry in Ca_V_1.2 demonstrated that the association of α_2_δ-1 increased the voltage sensitivity and pore-coupling of VSDs I–III (Savalli et al. 2016). The cryo-EM structure of Ca_V_1.1 and a mutagenesis study in Ca_V_1.2 revealed a prominent α_2_δ-1 binding site in the extracellular loop connecting S1 and S2 of VSD I (Wu et al. 2015; Bourdin et al. 2017). It is likely that this interaction modulates the intrinsic mechanism in VSD I responsible for the distinct activation kinetics of Ca_V_1.1 and Ca_V_1.2. Consequently, the diverse interactions of gating charges with countercharges of the ENC not only constitute the molecular basis for the great functional diversity of gating properties, but also may function as the points of action for the fine-tuning of these properties by various intrinsic and extrinsic mechanisms.

Similarly, to these modulators acting on the extracellular side of VSDs, binding of modulatory proteins on the intracellular side of ion channels might alter the delicate balance of gating charge–countercharge interactions and thereby modify the kinetics and voltage dependence of VSD state transitions. For example, binding of the G-protein βγ subunit to intracellular sites of PQ-type calcium channels inhibits this channel in a voltage-dependent manner (Currie 2010). Specifically, it converts the channel from a “willing” to a “reluctant” gating mode by shifting the voltage dependence of activation toward depolarized potentials. Interestingly, a recent voltage-clamp fluorometry study demonstrated that Gβγ binding to Ca_V_2.2 specifically inhibited the activation of VSD I and to a lesser degree that of VSD IV (Nilsson et al. 2024). This represents a striking example for the functional diversity among VSDs of a single channel and further demonstrates how the specific properties of individual VSDs are utilized for specific modulatory processes.

## Non-channel-gating functions of VSDs

Apart from their primary function in controlling the opening and closing of the channel gate, VSDs can serve other functions under physiological and pathological conditions. These non-channel-gating functions not only demonstrate the great functional versatility of VSDs, but also provide valuable insights into how structural modifications of the common building plan creates functional diversity.

### Skeletal muscle EC coupling

The prime example for such a non-channel-gating function of a VSD is the physiological role of Ca_V_1.1 in acting as voltage sensor of skeletal muscle excitation–contraction (EC) coupling. Since the 80s of the last century, it is clear that Ca_V_1.1 is the voltage sensor for skeletal muscle EC coupling and that this calcium-mediated signaling process is independent of Ca_V_1.1’s channel function (Rios and Brum 1987; Melzer et al. 1995). In short, Ca_V_1.1 controls the opening and closing of the SR calcium release channel (type 1 ryanodine receptor, RyR1) in response to membrane depolarization by a conformational coupling mechanism. Compared to this process, activation of the Ca_V_1.1 calcium channel requires much higher membrane potentials and the channel opens much too slowly to account for EC coupling. Recently, we reported that a single voltage sensor, VSD III, is responsible for this action (Pelizzari et al. 2024). In contrast, all four VSDs of Ca_V_1.1 contribute to the activation of the ion channel, albeit with distinct properties and functions. As discussed above, VSD I controls the speed of current activation and VSD IV limits the voltage dependence of activation in a splice-dependent manner. Also VSDs II and III need to be in the up-state to allow pore opening; however, their contribution to channel gating appears to be minor. This in itself is a striking example of functional diversity among the four VSDs of a single channel, and the structural basis of this diversity still needs to be fully resolved. However, this aspect is topped by the finding that the action of VSD III alone is linked to the conformational coupling of Ca_V_1.1 to the RyR1. VSD III’s voltage dependence near 0 mV and the comparatively rapid speed at which it responds to depolarization are sufficient to explain the specific activation properties of EC coupling compared to channel opening (which requires all four VSDs and is limited in speed and voltage dependence by VSD I and IV, respectively). Interestingly, molecular dynamics simulation of Ca_V_1.1 exposed to a membrane electric field suggest that VSD III undergoes non-canonical state transitions. In addition to the sliding-helix-like upward movement of S4, the other helices (S1–S3) undergo considerable vertical and lateral motions (Pelizzari et al. 2024). It is tempting to assume that these non-canonical movements initiate the conformational coupling to the RyR1.

### Omega (*ω*) currents and proton channels

Another phenomenon of VSDs not related to their primary function in activating channel gating is their ability to conduct ionic currents. The passage of the positive S4 gating charges through a pathway formed by the transmembrane helices S1–S3 corresponds to the conduction of immobilized ions across the membrane electric field. As discussed in detail above, this entails the translocation of the gating charges across the narrow seal formed by the HCS that is catalyzed by their intimate interactions with the phenylalanine and the two conserved countercharges of the CTC. In K_V_, Na_V_ and Ca_V_ channels disease mutations in the positions of the gating charges have been described that conduct proton or cation currents through this gating charge pathway of the mutated VSDs (Jurkat-Rott et al. 2012). Compared to the pore currents, these *ω* currents are small and they are state-dependent. In variants, in which inner gating charges (R3, R4, or R5) are mutated, *ω* currents occur when the membrane is depolarized. In variants, in which outer gating charges (R1, R2) are mutated, *ω* leak-currents appear at resting potentials. Apparently, when positioned in the CTC, the mutated amino acid breaks the seal of the HCS allowing protons and/or cations to pass through a continuous water channel (Monteleone et al. 2017). In fact, the appearance of *ω* currents in channels with mutated gating charges provided valuable evidence for the position of specific gating charges and the displacement of S4 upon depolarization, in support of the sliding helix model (Catterall et al. 2017). The ion selectivity of *ω* currents depends on the nature of the amino acid substituting the mutated gating charge. Histidine mutants generated proton currents, whereas substitution of the gating charges with less bulky uncharged amino acids generated cation currents. Interestingly, also histidine substitution of HCS residues in S1 and S2 resulted in proton *ω* currents (Campos et al. 2007). Together, these finding highlight the similarity of the gating charge transition pathway and a cation conduction pathway and demonstrate that substitutions of individual residues in the S4 or in the HCS can turn a VSD into a voltage-gated proton channel (Chanda and Bezanilla 2008).

Voltage-gated proton channels (H_V_1) are natural manifestations of such proton-conducting VSDs. They consist of a dimer of four helix bundles, each with a highly selective proton conduction pore and a bona fide positively charged S4 helix containing three gating charges (R1–R3) and an asparagine in the R4 position (Castillo et al. 2015). They also share with VSDs of ion channels the countercharges of the CTC in S2 and S3, and the phenylalanine in S2 marking the HCS (DeCoursey 2013). Like VSDs of conventional ion channels, they are gated by the outward movement of S4 in response to membrane depolarization. Their conductance is several orders of magnitude smaller than the conductance through the pores of tetrameric ion channels. Furthermore, the voltage dependence of activation is strongly modulated by the concentration of protons in the intracellular compartment. Altogether, their structure and functional properties strongly supports the notion that H_V_ channels correspond to the VSDs of K_V_, Na_V_ and Ca_V_ channels, modified in a way that allows them to function similar to channels with *ω* current mutations.

### VSD analogs in TRP channels

Above, we discussed how the functionally relevant interactions between the gating charges and countercharges of the ENC can act as sites for the modulation by various agents. From this, it is only a small step to VSDs, which activate channels primarily by chemical ligands or physical stimuli other than the membrane potential. The family of TRP channels essentially follows the tetrameric building plan of K_V_ channels with four subunits forming a central pore surrounded by four VSDs (S1–S4). Despite the presence of four VSDs TRP channels are not primarily gated by voltage, but by a host of physical and chemical stimuli (Talyzina et al. 2025). Consistent with the minor role of voltage in channel activation, the S4 helices of TRP channels contain few positive charges and even some negative charges. Also, the phenylalanine and the countercharges of the CTC are poorly conserved among the members of the TRP channel family (Palovcak et al. 2015). It is surprising that despite this paucity in potential gating charges, the channels still maintain some degree of voltage sensitivity. The structural basis and mechanisms by which the various physical and chemical stimuli activate TRP channel opening are only beginning to become unraveled. Nevertheless, several of them involve the function of the VSD. For example, co-activation of the cold-sensing TRPM8 by intracellular calcium and cooling agents requires their simultaneous association of these ligands to a binding pocket in the inner vestibule of the VSD (Huffer et al. 2024). Interestingly, amino acids important for ligand binding involve residues equivalent to gating charges and CTC countercharges in the VSDs of voltage-gated channels. Thus, it seems that the principle function of the VSD in controlling the channel gate is maintained in the TRP channels, whereas its function as sensor of voltage is adapted to other physical and chemical stimuli. The adaptations in a VSD necessary to confer temperature sensitivity onto a canonical voltage-gated ion channel have been elucidated by mutating in the shaker K_V_ channel amino-acid polarities at sites undergoing state-dependent changes in solvation (Chowdhury et al. 2014). Intriguingly, reducing S4 gating charges amplified the temperature sensitivity of the engineered channel. This provides strong support for a model of TRP channel gating proposing that in the temperature-sensitive TRP channels, heat and cold as well as agonists like capsaicin and menthol shift the voltage dependence of the channel activation toward physiological membrane potentials (Voets et al. 2004). Thus, TRP channels are impressive illustrations of how the built-in variability within the common building plan of VSDs is utilized to extend its function far beyond that of a voltage sensor.

## Distinct properties and functions of the four VSDs of Na_V_ and Ca_V_ channels

The examples described above clearly demonstrate that the four VSDs of pseudo-heterotetrameric ion channels differ with respect to structure, biophysical properties, and function. For example, in the skeletal muscle Na_V_1.4 channel, the fourth VSD activates more slowly than VSD I–III, and its transition into the up-state is not required for activation but for inactivation of the channel (Ahern et al. 2016) (Fig. 4). In the Ca_V_1.1 channel, VSDs I and IV activate at widely different voltages and with different kinetics, thus determining the characteristic properties of the skeletal muscle L-type calcium current (Flucher and Tuluc 2017). On the other hand, VSD III of Ca_V_1.1 activates fast and is the sole regulator of EC coupling (Pelizzari et al. 2024). In both cases, the distinct voltage dependencies and kinetics of the individual VSDs have been unambiguously demonstrated by the use of voltage-clamp fluorometry (Chanda and Bezanilla 2002; Savalli et al. 2021). These experiments further indicated the distinct contributions of the four VSDs to pore opening. In fact, voltage-clamp fluorometry data from Ca_V_ channels suggest that in these calcium channels, gating is dominated by only one or two of the VSDs (Pantazis et al. 2014; Savalli et al. 2021).

Conversely, this implies that functionally silent VSDs either do not respond to voltage changes at all, or that their state transition is not efficiently transmitted to the gate. For example, concurring evidence from protein structure determination and from voltage-clamp fluorometry analysis indicate that VSD II of the Ca_V_2 channels remains in the resting state when the cell membrane is depolarized (Gao et al. 2021; Yao et al. 2022b; Li et al. 2024; Nilsson et al. 2024). In contrast, the structures of Ca_V_1.1 and Ca_V_1.3 did detect VSD II in the up-state, and also voltage-clamp fluorometry of Ca_V_1.1 recorded signals for VSD II movement upon membrane depolarization (Wu et al. 2016; Savalli et al. 2021; Yao et al. 2022a; Bibollet et al. 2023). Interestingly, in a cryo-EM structure of Ca_V_1.2, VSD II was also captured in the resting state, whereas the fluorometry study revealed voltage-dependent state transitions for all four VSDs (Pantazis et al. 2014; Gao et al. 2023a). Consistent with a non-essential role of Ca_V_1.2 VSD II, channel function was not perturbed by the neutralization of all VSD II gating charges, while neutralization of the gating charges in the three other VSDs resulted in non-functional channels (Beyl et al. 2012). The notion that VSD II in Ca_V_ channels is of little or no importance for channel gating also extends to Ca_V_1.1. Several mutagenesis studies concur in observations, showing that mutations that cause considerable changes of gating properties in VSDs I, III, and IV are of little consequence in VSD II (Costé de Bagneaux et al. 2018; Pelizzari et al. 2024).

Together, these examples impressively demonstrate the diversity in structure and function of the four VSDs of pseudo-heterotetrameric ion channels. Stitching together, the four subunits/repeats of voltage-gated ion channels opened the door for structural and functional specialization of the VSDs. In this arrangement, the activation properties of a single VSD can limit the opening and closing of the channel gate in speed and voltage dependence. Conversely, other VSDs could become of lesser importance or functionally uncoupled from channel gating and even repurposed for non-channel functions. Phylogenetically, a large part of this specialization may have occurred before the separation of the channel subtypes, because many of these specializations are conserved across the members of the Ca_V_ and Na_V_ channel families (Groome and Bayless-Edwards 2020; Elhanafy et al. 2025); i.e., the structural and functional conservation is greater between the same VSDs of different channel subtypes than between the different VSDs of the same channel.

## Outlook

For our understanding of VSD function, their substantial structural and functional diversity has two major consequences: First of all, it teaches us that any mechanistic insight gleaned from the study of a single VSD may only apply to this specific VSD in the context of its channel. To be considered as general mechanism of VSD function, the same effects need to be demonstrated in a broader range of VSDs and channels. Second, the structural and functional diversity of VSDs creates new opportunities to study and understand the mechanisms ruling its functions. Comparing the structure, state transitions and biophysical properties of functionally distinct VSDs will reveal the structural basis of the functional specializations and thus the molecular mechanisms by which they regulate voltage dependence and kinetics of ion-channel gating.
